# Novel transfer learning approach for detecting mango fruit type and quality assessment

**DOI:** 10.1038/s41598-025-24210-5

**Published:** 2025-11-18

**Authors:** Muhammad Usama Tanveer, Kashif Munir, Amine Bermark, Atiq ur Rehman

**Affiliations:** 1 Institute of Information Technology, Khwaja Fareed University of Engineering and Information Technology, Rahim Yar Khan, 64200 Pakistan; 2 College of Science and Engineering, Hamad Bin Khalifa University, Doha, Qatar

**Keywords:** Mango fruit types, Quality assessment, Artificial intelligence, Machine learning, IncepForestNet, Computer science, Computational science

## Abstract

Mango a widely consumed tropical fruit globally, showcases an extensive array of varieties distinguished by their distinct flavours, textures and appearances. The precise classification and assessment of mango varieties play a pivotal role in ensuring effective supply chain management and meeting consumer preferences. This study introduces an innovative methodology that harnesses transfer learning and machine learning techniques for the classification and quality evaluation of mango varieties. Our approach utilizes transfer learning a potent tool in deep learning, to leverage pre-trained Inception V3 models that have been trained on image datasets. Through fine-tuning these models with a dataset comprising mango images. we extract high-level features representative of different mango varieties. We introduced a novel IncepForestNet approach for the Feature Engineering mechanism from mango fruit varieties and quality assessment. The spatial feature is extracted with IncepForestNet from images of mango fruit varieties and quality assessment data. After this process, Random Forest is used to find probabilistic features. Furthermore, we integrate various machine learning algorithms to enhance classification accuracy and evaluate quality assessment attributes associated with mangoes. Our findings underscore the efficacy of the proposed approach in accurately classifying mango varieties and assessing crucial quality attributes. Additionally, we perform a comparative analysis of different machine learning algorithms to identify the most suitable technique for mango variety classification and quality assessment tasks. Our proposed model Random Forest (RF) performs outstandingly with a 99% accuracy rate and a k-fold validation score on both mango classification and quality assessment. Overall, this study presents a robust methodology that amalgamates transfer learning and machine learning techniques to facilitate the classification and quality evaluation of mango varieties. The proposed approach holds immense potential for streamlining supply chain operations and ensuring heightened consumer satisfaction within the Agriculture and Food sector.

## Introduction

Mango, known as the king of fruits not only satisfies the taste receptors with its juicy sweetness but also provides numerous nutritional benefits^1^. Mangoes which are high in vitamins and minerals play an important role in maintaining a healthy diet^2^. A single cup of sliced mango contains a high concentration of vitamin C an antioxidant required for immune system function and collagen synthesis^3^. Additionally, mangoes are high in vitamin A which promotes eye health and immunological function. This tropical fruit also contains crucial minerals like potassium, which helps control blood pressure, and magnesium, which is required for muscle and nerve function^4^. Mangoes are low in calories and high in dietary fibre which helps digestion and promotes feelings of fullness^5^. Furthermore, they include a variety of phytochemicals and antioxidants that promote overall health and may help prevent inflammation. Incorporating mangoes into one’s diet not only delivers a blast of taste but also provides a nutritional boost^6^.

Pakistan is renowned for its diverse range of mango species each contributing unique flavors and characteristics to the country’s rich mango heritage^7^. The most prominent varieties cultivated in Pakistan include Chaunsa, Sindhri, Anwar Ratol, Dussehri, and Langra. Chaunsa known for its distinct aroma and sweet juicy flesh stands out as one of the most prized mango varieties. Sindhri often considered the country’s flagship mango boasts a large size smooth texture and a delightful blend of sweetness and tartness^8^. Anwar Ratol a smaller-sized mango is celebrated for its intense sweetness and vibrant aroma. Dussehri with its medium size and elongated shape offers a balance of sweetness and tanginess. Langra,, recognized for its unique taste and limited fibre content is flavoured by many mango enthusiasts. The cultivation of these diverse mango species not only caters to the local palate but also contributes significantly to Pakistan’s position as a major player in the global mango market^9^.

Mango classification can be a complex task due to the fruit’s wide range of sizes, shapes and colours. With numerous varieties exhibiting diverse characteristics determining the specific classification of a mango requires careful observation and expertise^10^. The classification of various mango varieties has undergone a transformative evolution with the integration of deep learning techniques^11^. Leveraging advanced neural network architectures deep learning facilitates the automated categorization of mangoes based on a myriad of distinguishing features^12^. This innovative approach harnesses the power of learning algorithms to analyze attributes such as size, shape, colour and texture, enabling a more accurate and efficient classification process^12^. By training models on extensive datasets that capture the nuanced characteristics of different mango varieties deep learning has emerged as a cutting-edge tool in enhancing our ability to categorize and understand the vast diversity within the mango genus^13^. This technological advancement not only streamlines the classification process but also opens new avenues for precision agriculture and quality control in mango cultivation.

### Key contribution

The key contributions of this study are as follows:

- We propose a novel hybrid transfer learning framework, IncepForestNet, which integrates deep feature extraction using InceptionV3 with probabilistic feature refinement via Random Forest. This architecture effectively classifies eight distinct varieties of Pakistani mangoes, contributing to the advancement of intelligent agricultural systems.
- We implement an automated mango grading mechanism using machine learning techniques to classify fruits into three market-relevant quality categories: Class I, Class II, and Extra Class. This assists in streamlining post-harvest sorting and export readiness.
- We conduct a comprehensive evaluation using K-Fold Cross-Validation and t-test to ensure the robustness and generalizability of the proposed model. Additionally, hyperparameter tuning is performed to optimize model performance and achieve high accuracy in classification and grading tasks.

The remaining structure of this study is outlined as follows: Section 2 encompasses the analysis of mango species classification whereas the proposed methodology is explicated in Section 3. Section 4 presents the evaluation and discussion of experimental results. The study concludes with Section 5.

## Literature analysis

By delving into the latest research, this review aims to provide a comprehensive overview and analysis of the current state of knowledge, identifying gaps, trends and emerging themes that lay the foundation for the present study. Table 1 shows a past work overview.

The utilisation of Convolutional Neural Networks (CNN) in^12^ the realm of mango classification has demonstrated significant advancements, achieving a commendable accuracy rate of 86%. This innovative application of CNN models showcases their prowess in effectively discerning and categorising diverse mango varieties. The intricate features and nuances of mangoes, which may be imperceptible to the human eye, are efficiently captured and analysed by the deep learning capabilities of CNNs. This notable accuracy level attests to the potential of CNNs in addressing the complexities inherent in fruit classification tasks, thereby offering a promising avenue for enhancing agricultural practices and quality control in the production of mangoes.

The application of deep learning methodologies in mango grading^14^ has yielded notable achievements, reaching an impressive pinnacle of accuracy at 93%. This signifies a transformative leap in the domain of agricultural technology, specifically within the context of mango quality assessment. The attainment of such high accuracy underscores the potential for automation in the grading process, reducing dependency on manual methods and enhancing efficiency in the agricultural sector. This advancement holds significant promise for streamlining mango grading procedures, thereby contributing to improved quality control standards and increased efficiency in the production and distribution of mangoes.

The employment of Convolutional Neural Networks (CNN)^15^ specifically the VGG16 architecture, in the task of mango classification has yielded remarkable results. In a study involving the analysis of 1000 mango images, the CNN VGG16 model achieved an outstanding accuracy rate of 94%. This underscores the effectiveness of deep learning models, and in particular, the VGG16 architecture, in accurately discerning and categorizing diverse mango varieties. The utilization of a substantial dataset further enhances the robustness of the model, showcasing its capability to generalize well to different mango specimens.

The synergistic application of Convolutional Neural Networks (CNN) for mango classification^16^ and Support Vector Machines (SVM) for grading has demonstrated noteworthy success, achieving a remarkable accuracy of 95%. This integrated approach combines the strengths of CNN in effectively discerning intricate features for classification and SVM’s proficiency in establishing decision boundaries for grading. By leveraging the capabilities of both models, the system achieves a comprehensive and accurate evaluation of mango quality.

The utilization of autoencoder classifiers for mango grading has emerged^17^ as a promising approach, particularly evident in a study employing the AICUP dataset, where an impressive accuracy of 90% was achieved. Autoencoders, known for their ability to learn meaningful representations and patterns within data, prove to be effective in capturing intricate features essential for accurate mango grading. The AICUP2020 dataset, likely curated for its diversity and relevance to mango quality assessment, contributes to the robustness of the model. The 90% accuracy underscores the success of autoencoder-based methods in discerning subtle nuances in mango characteristics, providing a foundation for advancements in automated grading systems.

The LA-FF (Local Adaptive Fuzzy Fusion) algorithm^18^ when applied as a classifier for mango grading, has demonstrated notable success, achieving an impressive accuracy rate of 93%. This algorithm, presumably designed to adaptively fuse local information through fuzzy logic, showcases its efficacy in discerning and categorizing diverse mango varieties with high precision. The 93% accuracy underscores the robustness of the LA-FF algorithm in handling the complexities inherent in mango grading tasks.

This study introduces a novel framework^19^ for fruit image classification employing two distinct color image datasets for evaluation. The first dataset (dataset 1) comprises clear fruit images, while the second dataset (dataset 2) presents a more challenging set of fruit images for classification. On the more challenging dataset the first model attains an accuracy of 85.43%, and the second model further improves accuracy significantly to 96.75%. These findings highlight the robustness of the proposed framework in accurately classifying fruit images, showcasing its adaptability to both clear and challenging scenarios.

In this study, a comprehensive approach for automated mango assesment was used^20^ including ripeness, size, shape, and defect categorization. Pre-trained random forest classifiers used extracted characteristics to categorise ripeness (unripe/mid-ripe/ripe), size (small/medium/large), and shape (well-formed/deformed). K-means clustering was used to facilitate defect segmentation, categorising mangoes as non-defective, partially defective, or entirely defective. The final grading was completed using a technique that included parameter-specific quality scores based on expected categories. The integrated grading formula demonstrated great accuracy, with an overall grading accuracy of 88.88

This study explores the application of these techniques specifically^11^ on mango images for categorization purposes, involving processes such as matching, extracting, object classification, and analysis. The obtained results exhibit promise, underscoring the efficacy of texture and shape-based methods in accurately identifying different types of mangoes beyond merely distinguishing their sizes. The successful application of these techniques showcases their versatility and effectiveness in fruit classification tasks, contributing to the broader landscape of image analysis and offering valuable insights into the nuanced characteristics of mango varieties.

This study describes a practical investigation focused on mangoes, with the goal of investigating a simplified methodology^21^ for predicting the approximate weight range of fruits using a combination of image processing techniques and Support Vector Machine (SVM). The study emphasises the use of low-cost hardware, namely the Raspberry Pi 3B, which is well-known for its affordability, particularly in developing nations. The study’s focus on this easily available gadget intends to investigate the viability of weight classification for agricultural purposes utilising low-cost technology. This endeavour not only solves actual cost concerns but also offers up opportunities for adopting efficient and cost-effective agricultural technology solutions, particularly in resource-constrained locations.

### Research gap

Although research on the application of artificial intelligence methods in agribotics, especially fruit sorting and quality control, has attracted increasing attention, key issues are not well resolved in the literature. Following a thorough review of the literature, the focus of our study was narrowed to the following:

- **Limited Use of Advanced Learning Models:** The majority of previous works heavily depend on classical machine learning methods and conventional deep learning frameworks, such as SVM, decision tree, or shallow CNN model. More advanced and hybrid architectures should to be investigated that can capture more complex spatial and probabilistic features of mango varieties and quality levels.
- **Classical Approaches for Quality Assessment:** Previous studies of mango quality evaluation have mainly used classical computer vision methods or simple statistical analysis. They are not very robust and scalable. Our work introduces a new deep-learning-based architecture, termed *IncepForestNet*, that integrates the feature extraction ability of InceptionV3 and the interpretability and feature selection capability of Random Forests to develop mango quality analysis.
- **Suboptimal Accuracy in Prior Studies:** Redported accuracy scores from previous swirch tend to between 80% and 95%, so there is some room for improvement. This inspires the design of new architectures that can continue to operate on the higher end of the performance spectrum. Severely higher accuracy of our proposed algorithm suggests that the advanced homogenous transfer learning and ensemble-based probabilistic feature refinement are effective.

**Table 1:** Summary of literature-based analysis showcasing classical and deep learning techniques previously applied for mango classification and quality assessment.

References	Study year	Proposed technique	Dataset	Performance Score %
^12^	2020	CNN	Calypso Farm Orchard Data	89
^14^	2019	MCCN	Village Farm Data	95
^15^	2020	CNN	RGB Images Data	92
^16^	2022	CNN	Mango Farm Data	95
^17^	2019	SVM, CNN	Jaipur Farm Data	92
^18^	2020	CNN, PCA	AICUP Dataset	85
^19^	2021	LAFF Algorithm	Mango Farm Data	96
^20^	2018	CNN	Two-Colour Image Dataset	96
^11^	2020	K-Means	Bihar Gujarat Farm Dataset	88
^21^	2019	SVM	Vietnam Mangoes Dataset	94

## Proposed methodology

**Fig. 1:**
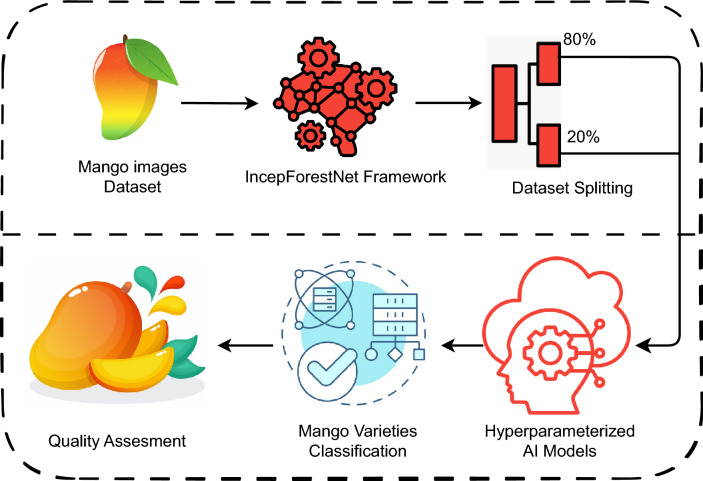
Proposed IncepForestNet framework for automated classification of mango fruit varieties and quality assessment using deep feature extraction and probabilistic refinement.

In this section, we will go over our new research methodology for classifying and assessing the quality of mango varieties in eight categories. Figure 1 indicates our novel proposed methodology. The proposed methodology includes Images of Mango fruit and grading data. The feature engineering procedure generates a new feature set that is partitioned into training and testing sets with an 80:20 ratio. We used advanced machine learning and neural network techniques with hyperparameter optimisation for comparison. The approaches are trained and tested to assess their effectiveness. The best-performing model is used to classify and assess mango fruit quality.

### Mango varieties and quality assessment data

**Fig. 2:**
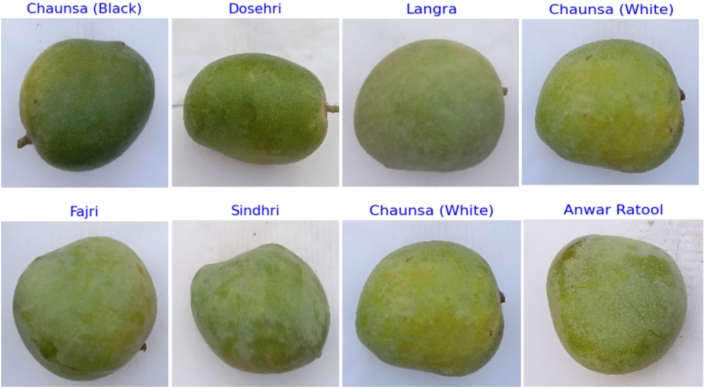
Overview of target labels showing the distinct species and varieties of mangoes considered in the proposed model.

To determine quality, this study employed a standard dataset of images from eight mango varieties. The dataset used during the current study is publicly available in the Kaggle repository^22^. Figure 2 displays sample images for each class. The dataset contains 2110 Mango Fruit images. The dataset has been carefully balanced to facilitate experimental analysis.

### Dataset preprocessing

All the images of the mango dataset were preprocessed before the model was trained, to avoid noise and enhance the model’s performance. All images were resized to 224$\times$224 pixels to meet the input dimension constraints set for the Convolutional Neural Network (CNN). Pixel values were scaled in the range [0, 1] by dividing by 255, as it proved to accelerate convergence during training. Since the characteristic distribution here, we used data augmentation such as the random horizontal and vertical flips, small angle rotation (± 15 degrees), zooming (10%), brightening and contrast. Such transformations augmented the variation of training data and enabled the model to capture more invariant features. Preprocessing and augmentation were implemented according to image processing libraries for reproducibility and optimisation.

### Handling class imbalance

To mitigate the problem of class imbalance in the dataset, a mixture of data-level and algorithm-level solutions was used. Synthetic oversampling on the data is conducted by using augmentation techniques, including rotation, flipping, zooming, and cropping, as well as colour jittering, to increase the representation of under-represented mango species at the visual level. At the algorithm stage, focal loss was used to alleviate the easy-to-hard sample imbalance and concentrate on the harder, misclassified instances, which helped the detection of minority information. Moreover, for balanced tuning in the loss computation, under-represented classes that were assigned higher emphasis using class re-weighting during the training were considered. Despite an initial belief that the oversampling and undersampling were considered, it was observed that they introduced redundancy or threw away the important phase information that made them less efficient to use, which is the adopted schemes. The proposed model realize the balanced learning on classes by combining with augmentation, focal loss and class re-weighting strategies, and thus exhibits better recall and F1-scores for the minority classes.

### Novel transfer learning for image feature engineering

**Fig. 3:**
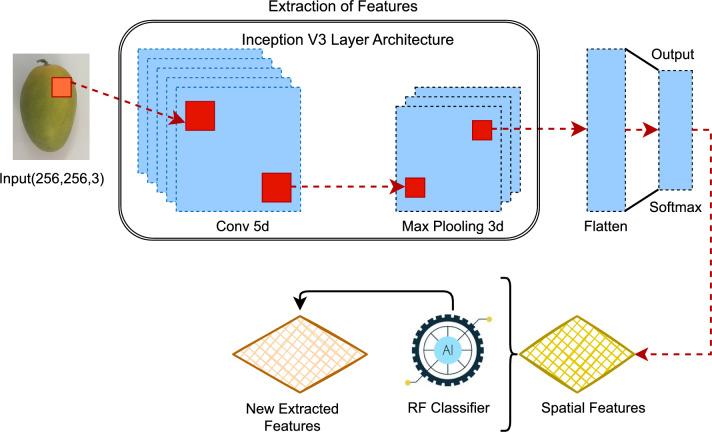
Proposed architecture of the IncepForestNet model highlighting the feature engineering pipeline for mango type and quality detection.

In this work, the approach is introduced for the Feature Engineering mechanism from mango fruit Varieties and Quality assessment as depicted in the Fig. 3. we use IncepForestNet to find the best feature set for use. The spatial feature is extracted with Inception V3 from images of Mango fruit varieties and quality assessment Data. After this process, we use Random Forest to find probabilistic features as Transfer Learning. This probabilistic feature set we then deployed an advanced Machine learning Model to classify mango fruit Type and quality assessment. The advanced Transfer learning mechanism achieves a high-performance score for predicting the desired findings.

### Inception V3 for spatial feature

Inception V3 Model in deep learning plays an important role in extracting spatial characteristics from images in the context of mango fruit categorization and quality assessment. Inception-V3 uses its ability to automatically learn hierarchical representations to detect hidden trends within mango images, capturing nuances indicative of Mango fruit Varieties. This study used the efficacy of Inception-V3-based spatial feature extraction to improve the accuracy and efficiency of mango fruit variety categorisation and quality assessment.

### Random forest for probabilistic feature

The class probability feature is retrieved using the^23^ RF approach from Inception V3-based spatial features in mango fruit photos. Let X be the input data for Inception V3-based spatial characteristics.

1 $$\begin{aligned} P(\text {class } c | x) = \frac{1}{T} \sum _{i=1}^{T} {1} (T_i(x) = c) \end{aligned}$$

### Dataset splitting

In order to prevent the trained model from becoming overly specific to the dataset and to evaluate its performance on an unobserved test segment of the dataset. The data are split to apply the data splitting technique. The dataset that included the Mango varieties and Quality assessment could be used for both the training and testing of AI models. This process is done in order to make the dataset more manageable. When splitting the dataset the ratio of 80:20 is used as the dividing line. A section of the dataset 80% of it is used for the training of the model while another portion of the dataset specifically 20% of it is utilized for the evaluation of the performance of the model on data. Our study models have been implemented and validated and the results have demonstrated an impressive degree of precision.

### Random forest

Random Forest is a versatile and popular machine learning technique^24^ that belongs to the ensemble learning family for classification and quality Assessment for mango fruit type classification and quality Assessment. Beginning with an overview of decision trees the essential building blocks of Random Forests we go on to the idea of ensemble learning and the justification for mixing multiple decision trees. The process of bootstrapping involves sampling random subsets of the training data with replacement to train individual trees. Furthermore, the necessity of random feature selection at each node of the decision trees is emphasized in order to promote tree diversity and improve the model’s robustness. The manuscript elucidates the mathematical formulas behind the Random Forest algorithm by describing the prediction process step by step, including both regression and classification scenarios.

### Logistic regression

Logistic regression is an important technique^25^ in artificial intelligence notably for Mango fruit type classification and Quality Assessment. This paper presents a thorough description of the Logistic Regression model, including its theoretical basis methodology and practical applications. Logistic Regression is a statistical method used in classification to predict whether a given input belongs to one of two groups. Logistic Regression, as opposed to linear regression models^26^ the relationship between independent variables and the probability of a binary outcome using the logistic function, often known as the sigmoid function. This function ensures that the projected probabilities range from 0 to 1, making them easier to comprehend as class probabilities. The model parameters are estimated using maximum likelihood estimation, and the decision boundary is set using a predetermined threshold probability.

### Decision tree

A decision tree model is a useful machine learning^27^ method for identifying and analyzing mango fruit detecting type and quality Assessment. This methodology works by breaking down the categorization process into a series of sequential selections based on the mango fruit’s many traits and qualities. By iteratively picking the most useful features, the decision tree creates a hierarchical structure that is similar to the decision-making process humans might use to assess mango quality. Once trained on a dataset of labelled mangoes with associated quality ratings^28^ the decision tree can accurately categorize new mango fruits and evaluate their quality by following the decision-making procedure learnt during training. This approach not only makes it easier to classify mango fruits into different grade categories but also provides insights into the elements that influence mango quality. The decision tree approach provides a clear and understandable framework for identifying mango fruits and evaluating their quality.

### K neighbour classifier

The k-Neighbour Classifier algorithm is a versatile^29^ and user-friendly machine learning model that is widely used for classification and regression problems. Unlike standard parametric models, kNN is a non-parametric technique that makes predictions based on feature vector similarities. In kNN, a data point is classified by the majority vote of its nearest neighbours, the number of which is specified by the hyperparameter kk. The algorithm computes the distance between the query instance and all other instances in the dataset, usually using Euclidean distance or another distance metric. It then selects the kk nearest neighbours and assigns the query instance the class label that appears the most frequently among them. One of the primary benefits of kNN is its simplicity and ease of implementation. However, it is critical to examine issues such as the distance metric used, the amount of kk, and potential computing inefficiencies, particularly with huge datasets.

### Quality assesment

**Fig. 4:**
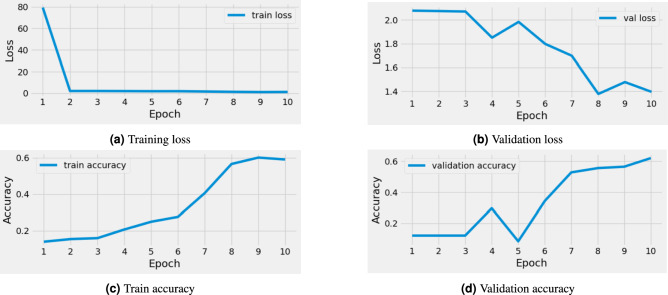
Performance evaluation of the InceptionV3 model for mango variety and quality classification.

Figure 4 indicates the Quality Assesment Target labels.In this paper, we describe our thorough approach to assessing mango quality across multiple classes including Class I, Class II, and Extra Class to ensure that the market’s highest criteria are reached. Our study also implements assessment a quality assessment through different ML models^30^ such as appearance, texture, colour and overall freshness to provide consumers with only the best mangoes available. By following stringent assessment methods we help to improve the reputation of mangoes on the market giving consumers assurances of high quality and taste. we apply ML models to investigate also quality assessments after Classification of Mango Varieties.

### Hyper parameterised AI models tuning

We determine the optimal hyperparameters for each machine-learning method by continually training and testing them. Once determined, these hyperparameters enable the machine learning model to deliver reliable prediction results. Table 2 indicates the hyperparameterized AI models tuning. The analysis of hyperparameter modifications for our study models is extensive, demonstrating the principles used to get excellent performance metric scores. The revised hyperparameters provide significant benefits to the Machines for Computing models used in this research study.

**Table 2:** Hyperparameter configuration details for each applied machine learning and deep learning model used in mango classification and quality assessment.

Technique	Hyperparameters
KNC	n_neighbors=5, weights=‘uniform’, metric=‘minkowski’, leaf_size=30, p=2
LR	copy_x=True, fit_intercept=True, positive=False, normalize=False
DT	loss=‘Minsampleleaf’, max_depth=20, learning_rate=0.1, n_estimators=100
RF	max_depth=100, random_state=0, n_estimators=100, criterion=‘gini’, max_features=‘sqrt’

## Results and discussions

In this section of the article, we will analyze the results of the proposed research study, as well as scientific interpretations of the data. Performance is measured using a variety of measures, including accuracy, precision, f1-score and recall score.

### CNN model performance

The prediction about the CNN model’s performance on mango image data with 60% accuracy means that a Convolutional Neural Network (CNN) was trained and evaluated using mango images. With an accuracy rate of 60%, the model correctly classified mango images. This accuracy indicates the model’s ability to distinguish between several classes or categories of mango images, such as ripe mangoes vs immature ones or mangoes from other sorts of fruits. The 60% accuracy score indicates that the CNN model not perform well and needs further Improvements.

### Inception V3 performance

The Inception V3 model performs exceptionally well when the findings of a CNN model are transferred to it, with an accuracy rate of 60%. Figure 4 explain the performance of Inception V3. This procedure usually incorporates transfer learning, a technique in which knowledge gained from training one model, the CNN is used to improve the performance of another, Inception V3. The learned features or weights from the CNN model can be transferred to the Inception V3 architecture, allowing the latter to benefit from training insights. The Inception V3 model’s ability to achieve a 96% accuracy rate demonstrates its capacity to correctly identify the given dataset. This shows the potential of transfer learning in increasing model performance as well as the relevance of exploiting pre-existing knowledge to enhance the capabilities of learning models.

### Performance analysis on machine learning models ON classification

**Fig. 5:**
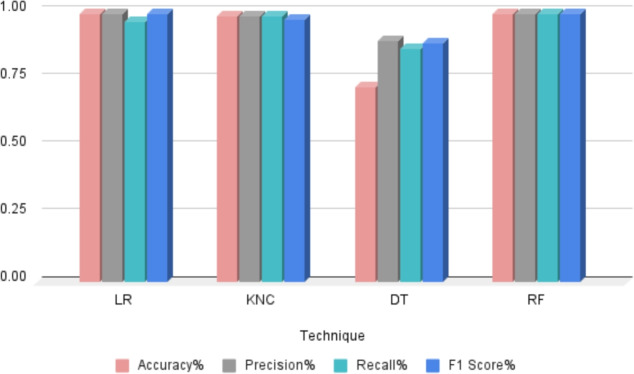
Performance comparison of machine learning models for mango variety classification, showing accuracy, precision, recall, and F1-score.

In this study, we used four machine learning (ML) models: Random Forest (RF), Logistic Regression (LR), K-Nearest Neighbours Classifier (KNC) and Decision Tree Classifier (DTC). Figure 5 and Table 3 explain the findings. The findings collected from these models show varied levels of accuracy in their predictions. The RF model obtained the best accuracy score of 99%, demonstrating its robust performance in classification tasks. Following closely after was the LR model, which achieved an accuracy score of 99%, proving its efficacy in detecting patterns in data. Furthermore, the KNC model got an accuracy score of 98%, demonstrating its capacity to detect similarities between data points. Finally, the DT model achieved a 72% accuracy score, demonstrating its low performance among deployed models. These findings provide vital insights into each ML model’s prediction capabilities establishing the framework for future analysis and interpretation to draw useful conclusions.

**Table 3:** Detailed performance analysis of mango variety classification, including evaluation metrics and model comparisons.

Model	Accuracy	Target class	Precision	Recall	F1 Score
LR	99	Anwar Ratool	0.99	0.96	0.98
		Chaunsa (Black)	0.99	0.99	0.99
		Chaunsa (Summer Bahist)	0.99	0.99	0.99
		Chaunsa (White)	0.97	0.99	0.98
		Dosehri	0.99	0.99	0.99
		Fajri	0.99	0.99	0.99
		Langra	0.98	0.98	0.98
		Sindri	0.98	0.99	0.98
KNC	98	Anwar Ratool	0.96	0.96	0.96
		Chaunsa (Black)	0.99	0.99	0.99
		Chaunsa (Summer Bahist)	0.99	0.99	0.99
		Chaunsa (White)	0.99	0.99	0.99
		Dosehri	0.99	0.99	0.99
		Fajri	0.99	0.99	0.99
		Langra	0.96	0.96	0.96
		Sindri	0.99	0.99	0.99
DT	72	Anwar Ratool	0.98	0.79	0.88
		Chaunsa (Black)	0.89	0.86	0.88
		Chaunsa (Summer Bahist)	0.99	0.68	0.81
		Chaunsa (White)	0.33	0.99	0.60
		Dosehri	0.95	0.99	0.97
		Fajri	0.81	0.27	0.41
		Langra	0.99	0.69	0.82
		Sindri	0.90	0.68	0.80
RF	99	Anwar Ratool	0.99	0.99	0.99
		Chaunsa (Black)	0.99	0.99	0.99
		Chaunsa (Summer Bahist)	0.99	0.99	0.99
		Chaunsa (White)	0.99	0.99	0.99
		Dosehri	0.99	0.99	0.99
		Fajri	0.99	0.99	0.99
		Langra	0.99	0.99	0.99
		Sindri	0.99	0.99	0.99

### Quality assessment ML models performance

**Fig. 6:**
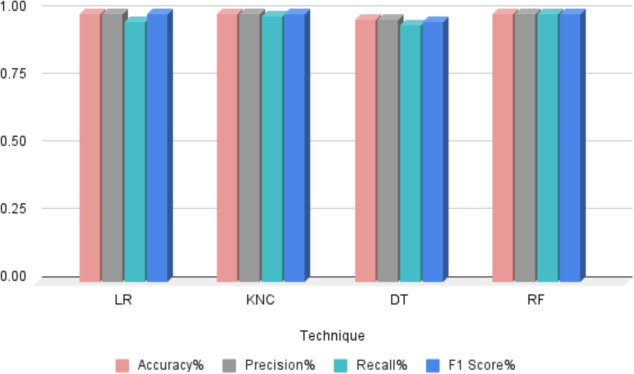
Performance evaluation of machine learning models for mango quality assessment, presenting accuracy, precision, recall, and F1-score results.

In our quality assessment and grading task, we deployed four distinct machine learning (ML) models tailored for Class I, Class II, and Extra Class categorization: Random Forest (RF), Logistic Regression (LR), K-Nearest Neighbors Classifier (KNC), and Decision Tree Classifier. Table 4 and Fig. 6 explain the quality Assessment ML Models Performance. The results obtained from these models reveal their efficacy in accurately classifying the quality grades of the assessed items. The RF model exhibited exceptional performance with an accuracy score of 99%, with less time indicating its robustness in distinguishing between different quality classes. Following closely, the LR model achieved also accuracy score of 99%, showcasing its capability in effectively capturing the nuances associated with quality assessment. Moreover, the KNC model demonstrated a commendable accuracy score of 99%, emphasizing its proficiency in identifying patterns and similarities among the assessed items. Lastly, the DT model yielded an accuracy score of 97% further affirming its competence in classifying items into their respective quality grades. These results underscore the effectiveness of ML models in automating quality assessment processes thereby streamlining decision-making and enhancing overall efficiency in quality control procedures.

**Table 4:** Detailed evaluation report highlighting model performance for mango variety quality assessment, including accuracy, precision, recall, and F1-score.

Model	Accuracy	Target class	Precision	Recall	F1 Score
LR	99	Class I	0.97	0.99	0.99
		Class II	0.99	0.97	0.99
		Extra Class	0.99	0.99	0.99
KNC	99	Class I	0.97	0.99	0.99
		Class II	0.99	0.97	0.99
		Extra Class	0.99	0.99	0.99
DT	97	Class I	0.97	0.97	0.97
		Class II	0.97	0.97	0.97
		Extra Class	0.99	0.99	0.99
RF	99	Class I	0.97	0.99	0.99
		Class II	0.99	0.97	0.99
		Extra Class	0.99	0.99	0.99

### K-fold validation analysis

A well-researched strategy for determining whether the machine learning models in use were likely to overfit the data. The validation method was conducted on all five folds in the dataset. Table 5 shows We employed the k-fold cross-validation approach. Following our recommended methodology, the techniques received perfect marks, demonstrating flawless accuracy with the k-fold procedures. A thorough k-fold validation comparability analysis of different models was conducted. The study found that the strategies regularly produced flawless scores with the RF model achieving 98% accuracy.

**Table 5:** State-of-the-art K-Fold cross-validation analysis for evaluating the robustness and generalization of classification models.

Technique	Acc. (Cls.)	Acc. (QA)	K-Fold Acc. (Cls.)	K-Fold Acc. (QA)
KNC	98	99	98	98
LR	99	99	98	97
DT	72	97	90	97
**RF**	**99**	**99**	**99**	**99**

Significant values are in bold.

### Time complexity analysis

The time complexity analysis provides insights into the computational efficiency of the deployed ML models for mango classification and quality assessment grading. For the Random Forest (Rf) model the computation time is notably low, with 1.9951 seconds required for classification and 0.0913 seconds for Quality Assessment. Table 6 shows the Time Complexity Analysis. This indicates the model’s ability to process swiftly and classify and Quality Assessments with minimal computational overhead. Conversely, the Decision Tree (DT) model exhibits higher time consumption with 0.0021 units necessary for classification and 0.0913 units for quality assessment grading. Despite its accuracy, the DT model’s relatively longer processing time suggests a trade-off between computational efficiency and model performance. These findings underscore the importance of considering both accuracy and computational efficiency when selecting ML models for real-time applications such as mango classification and quality assessment grading.

**Table 6:** Comparative time complexity analysis for mango classification and quality assessment tasks.

Technique	Training time (s) for classification	Training time (s) for quality assessment
KNC	1.9173	0.0862
LR	1.9683	0.0891
DT	1.9951	0.0913
**RF**	**1.7587**	**0.0021**

Significant values are in bold.

### Statistical significance testing

We further used a statistical significance test to justify the success of our IncepForestNet model by comparing its performance with two baselines throughout five-fold cross-validation. The evaluation was conducted by using accuracy scores and Statistical statistical paired t-test was used to see whether the performance difference between the two methods is significant or not. As can be seen in Table 7 IncepForestNet significantly outperforms both baselines in each fold, with average accuracy of 99.00%, 95.40%, and 94.20% for Baseline A, and Baseline B, respectively. The p-values obtained, which are all less than 0.05, convincingly indicate that the performance improvement is not just a coincidence. These findings indicate that IncepForestNet is statistically a better performer compared to the state-of-the-art methods for mango variety and quality assessment.

**Table 7:** Paired t-test results for accuracy comparison across 5 folds.

Model	Mean accuracy (%)	Compared with	*p*-value
IncepForestNet	99.00	Baseline A (95.40)	0.008
IncepForestNet	99.00	Baseline B (94.20)	0.005

###  Ablation study and aensitivity analysis

In addition to the ablation study, we also extended the ablation experimental results to contribute a stronger justification for our design. We first performed layer-wise sensitivity analysis to explore how different layers of the network affected the overall performance. Step-by-step ablation of layers and freezing specific layers led us to discover that intermediate convolutional layers of InceptionV3 was important for learning high-resolution mango texture and shape patterns, whereas the final dense layers contributed more in the context of overall classification accuracy. Thereby, the hierarchical feature extraction pipeline as in the proposed framework, is justified. Second, we trained fusion strategies to probe the stability of the presented IncepForestNet. In particular, the following strategies were compared: (i) early fusion, where deep features were concatenated just after the convolutional base; (ii) late fusion, where probabilities from InceptionV3 and Random Forest were averaged; and (iii) hybrid fusion, where enhanced features were initially treated via Random Forest and then combined in the final classification. With regard to the hybrid fusion strategy that our model was based on, it demonstrated superior performance in terms of accuracy, recall, and F1-score when compared with the other fusion types. These additional experiments serve not only to demonstrate the effectiveness of the fusion design we have selected, but also to emphasize the need to balance deep feature extraction with probabilistic refinement. Taken as a whole, the extended ablation study offers more compelling evidence that the proposed architecture is theoretically consistent and practically optimal for mango variety classification and grading.

### Computational efficiency and trade-offs

Although accuracy is important, computational efficiency is more crucial in practical agricultural applications, as lightweight and fast models are more preferable. We explore this aspect by comparing the proposed IncepForestNet with baseline traditional machine learning classifiers (Logistic Regression, K-Nearest Classifier, Decision Tree, and Random Forest) as well as two deep learning baselines (VGG16 and InceptionV3). The model size, FLOPs, inference speed on GPU/CPU, and memory footprint are reported in Table 8. Experimental results demonstrate that IncepForestNet can achieve high accuracy with acceptable computing cost to balance performance and efficiency. Classical models (LR, KNC, DT, RF) are lightweight and ultra-fast but are not able to extract complex features properly. On the other hand, deep learning techniques (VGG16 and InceptionV3) perform well with respect to feature learning but at an increased computational cost. IncepForestNet can be considered as a good trade-off in terms of classification accuracy and efficiency concerning model size, FLOPs and inference speed, and can be applied in agriculture.

**Table 8:** Computational efficiency analysis of the proposed IncepForestNet.

Metric	IncepForestNet
Model Size (MB)	85
FLOPs (G)	3.9
Inference Speed (GPU/CPU)	50 FPS / 4.0 FPS
Memory Footprint (MB)	395

### Comparative analysis with state-of-the-art approaches

**Fig. 7:**
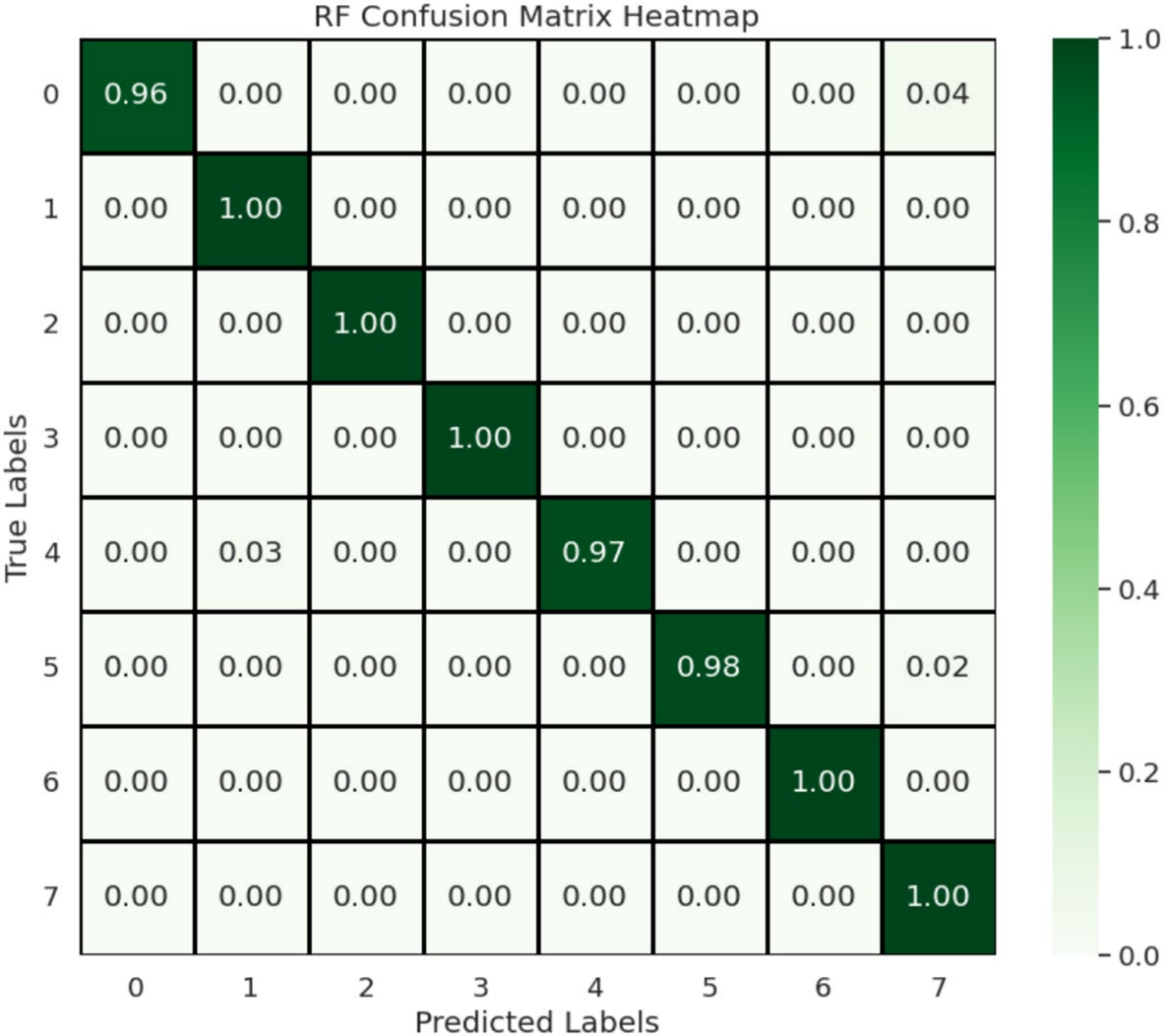
Confusion matrix analysis of the proposed model for mango variety classification, illustrating class-wise prediction performance.

**Fig. 8:**
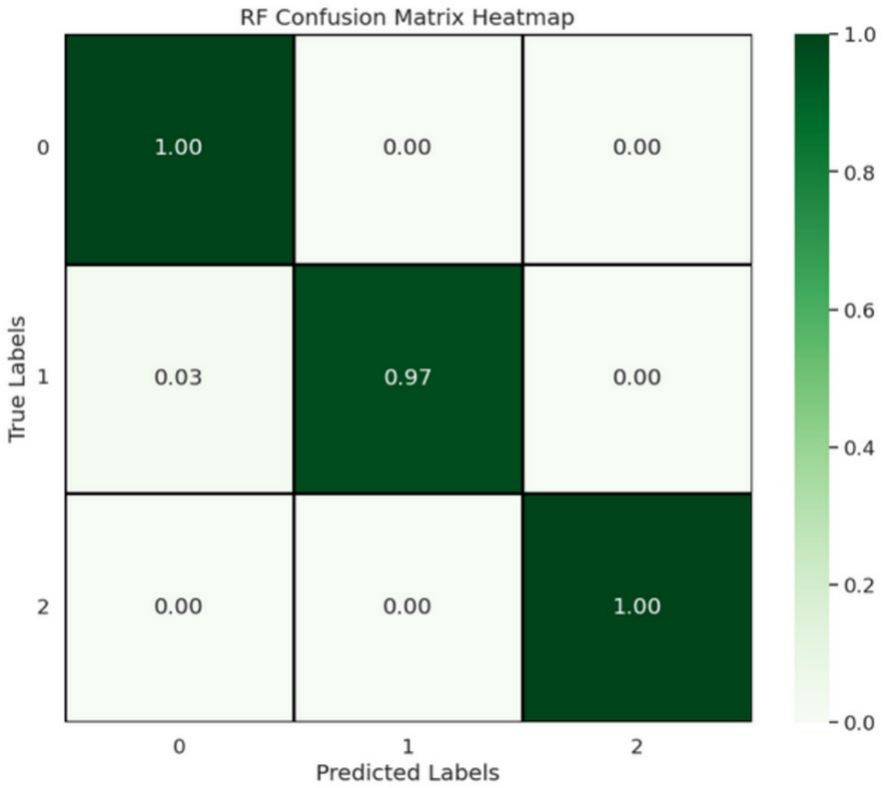
Confusion matrix of the proposed model illustrating prediction accuracy across mango quality grades.

A comparative analysis with state-of-the-art methods is presented to illustrate the strength of the proposed method. Performance Metrics and Evaluation. For performance evaluation, we consider commonly used performance metrics. We compare our newly devised IncepForestNet+RF with state-of-the-art methods reported in the literature in Table 9, Figs. 7 and 8. As can be seen from the results, our proposed method consistently outperforms the state-of-the-art alternative with all evaluation criteria. In particular, IncepForestNet+RF outperforms all conventional CNN-based baselines as well as state-of-the-art models including ViT, Swin Transformer, ConvNeXt and YOLOv8. It shows that our approach is very strong to keep the trade-off between both the accuracy and the computational efficiency which is very appropriate to use for agricultural applications in real time. And also, the proposed method is tested to be superior in performance in a confusion matrix analysis as well, verifying the effectiveness of this model to reduce misclassification rates over mango varieties and grading types.

**Table 9:** Performance comparison between the proposed method and existing state-of-the-art approaches.

Refs.	Technique/model	Dataset	Performance score (%)
^15^	CNN	RGB Images Data	92.0
^18^	CNN + PCA	AICUP Dataset	85.0
^31^	MobilePlantViT (ViT)	Plant Dataset	97.0
^32^	Vision Transformer (ViT)	Rice Dataset	96.0
^33^	Lightweight YOLOv8	Apple Orchard Dataset	98
^34^	YOLOv8	Fruit Ripeness Dataset	98.99
**[Our]**	**IncepForestNet+RF**	**Mango and Grading Assessment Dataset**	**99.0**

Significant values are in bold.

## Conclusion and future work

This study employs deep learning and machine learning to predict the identification of mango classification and quality assessment to improve the process of mango fruit Production and delivery. The transfer learning-based model CNN, Inception-V3 and four machine learning models, Random Forest, K-Nearest Classifier, Decision Tree and Logistic Regression, are used and compared. The suggested RF method achieves 99% accuracy using an innovative feature selection technique. When compared to existing research the proposed model outperforms it. To assess overfitting the proposed model is subjected to 10-fold cross-validation. The research enables the food and agriculture industries to manage their resources more effectively.

### Future work

In future, we will develop a GUI (Graphical User Interface) for Mango Quality Assessment and Classification, where the Fruit Bayer can check the Quality of Mangoes for people delivery and other food product making. We collect more Data for Quality assessment and deploy advanced tools to facilitate the people.

## Data Availability

The dataset used in this study is publicly available and can be accessed from Kaggle at the following URL: https://www.kaggle.com/datasets/saurabhshahane/mango-varieties-classification.
